# Expression and Regulation of Nampt in Human Islets

**DOI:** 10.1371/journal.pone.0058767

**Published:** 2013-03-11

**Authors:** Karen Kover, Pei Ying Tong, Dara Watkins, Mark Clements, Lisa Stehno-Bittel, Lesya Novikova, Doug Bittel, Nataliya Kibiryeva, Jacob Stuhlsatz, Yun Yan, Shui Qing Ye, Wayne V. Moore

**Affiliations:** 1 Section of Endocrine/Diabetes, Children's Mercy Hospital and University of Missouri-Kansas City School of Medicine, Kansas City, Missouri, United States of America; 2 Department of Physical Therapy & Rehabilitation Sciences, University of Kansas Medical Center, Kansas City, Kansas, United States of America; 3 Section of Genetics Research, Children's Mercy Hospital and University of Missouri-Kansas City School of Medicine, Kansas City, Missouri, United States of America; University of Padova, Medical School, Italy

## Abstract

Nicotinamide phosphoribosyltransferase (Nampt) is a rate-limiting enzyme in the mammalian NAD+ biosynthesis of a salvage pathway and exists in 2 known forms, intracellular Nampt (iNampt) and a secreted form, extracellular Nampt (eNampt). eNampt can generate an intermediate product, nicotinamide mononucleotide (NMN), which has been reported to support insulin secretion in pancreatic islets. Nampt has been reported to be expressed in the pancreas but islet specific expression has not been adequately defined. The aim of this study was to characterize Nampt expression, secretion and regulation by glucose in human islets. Gene and protein expression of Nampt was assessed in human pancreatic tissue and isolated islets by qRT-PCR and immunofluorescence/confocal imaging respectively. Variable amounts of Nampt mRNA were detected in pancreatic tissue and isolated islets. Immunofluorescence staining for Nampt was found in the exocrine and endocrine tissue of fetal pancreas. However, in adulthood, Nampt expression was localized predominantly in beta cells. Isolated human islets secreted increasing amounts of eNampt in response to high glucose (20 mM) in a static glucose-stimulated insulin secretion assay (GSIS). In addition to an increase in eNampt secretion, exposure to 20 mM glucose also increased Nampt mRNA levels but not protein content. The secretion of eNampt was attenuated by the addition of membrane depolarization inhibitors, diazoxide and nifedipine. Islet-secreted eNampt showed enzymatic activity in a reaction with increasing production of NAD+/NADH over time. In summary, we show that Nampt is expressed in both exocrine and endocrine tissue early in life but in adulthood expression is localized to endocrine tissue. Enzymatically active eNampt is secreted by human islets, is regulated by glucose and requires membrane depolarization.

## Introduction

Nicotinamide phosphoribosyltransferase (Nampt) is a rate-limiting enzyme in the mammalian NAD+ biosynthesis of a salvage pathway and exists in 2 known forms, intracellular Nampt (iNampt) and a secreted form, extracellular Nampt (eNampt). This enzyme has been shown to have a variety of physiological functions depending on the pathophysiological conditions and type of tissues studied. eNampt is also known as both pre-B cell colony-enhancing factor (PBEF) due to its function as a cytokine and visfatin due to its role as an adipokine. It may also act as an extracellular enzyme converting extracellular nictotinamide to nicotinamide mononucleotide (NMN). NMN, an intermediate product in NAD+ biosynthetic pathway, may be taken up by cells and utilized to generate NAD+/NADH [1]–[3]. Besides extracellular enzymatic activity, eNampt has reportedly been shown to function in a non-enzymatic capacity by activating receptors in several cell types. There are several reports that eNampt activates/binds to insulin receptors resulting in signal transduction in adipocytes, osteoblasts and pancreatic beta cell [3]–[5].

The role of Nampt in metabolic dysfunction such as diabetes and obesity is not well defined. In some cases a positive correlation has been made with increasing plasma/serum levels of Nampt and type 1 and 2 diabetes [6]–[10]. However, others report opposite findings with decreasing or no changes in plasma/serum Nampt levels associated with diabetes/obesity [11]–[16]. The conflicting results may be due, in part, to the types of populations studied, small sample size, and/or variability in the types of assays used to measure serum/plasma eNampt [17]. The role of eNampt in maintaining normal metabolic responses has been demonstrated using rodent models. In a heterozygous NAMPT knockout (KO) mouse model (NAMPT+/−), it was reported that decreased NAMPT expression resulted in glucose intolerance in females and impaired glucose-stimulated insulin release by isolated islets [1]. Interestingly, treating KO mice or isolated islets with NMN restored glucose tolerance and insulin secretion. The role of NAMPT in insulin sensitivity and lipid metabolism was demonstrated in a rat model that over expressed NAMPT. These rats showed improved insulin sensitivity and lipid profiles on day 4 after injection of plasmids [18]. Another report described the protective effects of eNampt via NMN treatment in restoring mouse beta cell function after exposure to pro-inflammatory cytokines such as IL-1beta and TNFalpha [19]. Taken together these reports suggest that eNampt/NMN has an important role in maintaining beta cell function and survival.

There is an assumption that beta cells must rely on enzymatic activity of circulating eNampt to generate NMN due to the lack of islet specific Nampt expression/secretion. The characterization of Nampt expression/secretion in human islets has not been adequately investigated. The aim of this study is to characterize islet specific Nampt expression, secretion and regulation by glucose in human islets.

## Methods

### Tissue Source and Ethical Statements

Isolated islets were obtained from Axon Cells formally known as BetaPro LLC (Gordonsville, VA). Pancreas procurement for islet isolation was approved by University of Virginia Institutional Review Board for Health Sciences Research Committee. Pancreatic tissue was obtained from 3 sources; BetaPro LLC (Gordonsville, VA), University of Kansas Pathology (KU Path, Kansas City, KS) and National Institute of Childhood Diseases Brain and Tissue bank for Developmental Disorders at the University of Maryland, Baltimore MD, contract HHSN275200900011C, ref. no. N01-HD-9-0011 (BTB). Written ethical approval was obtained from the following committees: University of Virginia Institutional Review Board for Health Sciences Research (BetaPro LLC); University of Kansas Medical Center Human Research Protection Program (KU Path); University of Maryland Institutional Review Board (BTB). In all cases informed written consent was obtained from all donors or donor relatives. See table 1 for donor demographics.

**Table 1:** Donor Demographics.

*Tissue Type*	*Source*	*Age*	*Race*	*Gender*	*BMI*
Islets & pancreas	BetaPro LLC	39yr	Caucasian	Fe	40
Islets	BetaPro LLC	59yr	Caucasian	Fe	28.2
Islets	BetaPro LLC	58yr	African Am	Fe	29.5
Islets	BetaPro LLC	51yr	n/a	M	28.4
Islets	BetaPro LLC	54yr	African Am	Fe	53.1
Islets	BetaPro LLC	43yr	Caucasian	Fe	33.9
Islets	BetaPro LLC	35yr	Caucasian	Fe	32
Islets	BetaPro LLC	26yr	African Am	Fe	33
Islets	BetaPro LLC	44yr	Caucasian	M	30.4
Islets	BetaPro LLC	58yr	Caucasian	Fe	20
Islets	BetaPro LLC	59yr	Caucasian	M	n/a
Islets	BetaPro LLC	50yr	African Am	Fe	36.3
Islets	Axon Cells	51yr	Caucasian	M	32
Islets	Axon Cells	48yr	African Am	Fe	26.8
Pancreas	BTB	22wk gestation	Hispanic	Fe	n/a
Pancreas	KU Path	50yr	n/a	M	n/a
Pancreas	BTB	2.8yr	n/a	Fe	n/a
Pancreas	BTB	19wk gestation	Caucasian	M	n/a
Pancreas	BTB	3yr	African Am	Fe	n/a
Pancreas	KU Path	72yr	n/a	M	n/a
Pancreas	BTB	125 days	Caucasian	Fe	n/a
Pancreas	BTB	141 days	African Am	M	n/a
Pancreas	BTB	66 days	Caucasian	Fe	n/a
Pancreas	BTB	12yr	Caucasian	Fe	n/a
Pancreas	BTB	18yr	Caucasian	Fe	n/a
Pancreas	KU Path	82yr	Caucasian	Fe	n/a
Pancreas	BTB	6yr	Caucasian	Fe	n/a
Pancreas	BTB	53yr	Caucasian	Fe	n/a
Pancreas	BTB	48yr	Caucasian	Fe	n/a
Pancreas	BTB	42yr	Caucasian	Fe	n/a

KU Path, University of Kansas Pathology; BTB, National Institute of Childhood Diseases Brain and Tissue bank for Developmental Disorders at the University of Maryland, Baltimore MD; n/a, not available.

### Gene expression

Nampt expression was analyzed by quantitative Real Time-PCR (qRT-PCR) using a NAMPT specific Taqman assay (Applied Biosystems/Life Technologies, Carlsbad, CA.) according to the manufacturer's instructions. Briefly, 100ng of total RNA from each sample was reversed-transcribed using the SuperScript III First-Strand Synthesis SuperMix for qRT-PCR (Life Technologies, Carlsbad, CA) according to the manufacturer's protocol. This was followed by RT-PCR amplification in triplicate using an ABI 7000 sequence detection instrument. The point at which the intensity level crossed the PCR cycle threshold (C_T_) was used to compare individual reactions. Normalization of the qRT-PCR reactions used the 2^(−ΔΔCT)^ method with GAPDH as the standardization gene for each sample to correct for minor experimental error as reported previously [20], [21]. Normalized C_T_ values were averaged to produce the mean C_T_ value.

### Immunohistochemistry

Paraffin-embedded 8 um thick pancreatic tail sections from human donors were deparaffinized/rehydrated in xylene followed by ethanol and phosphate-buffered saline (PBS), pH 7.4 using standard procedures. Antigen was retrieved using a steamer (30 min) in Shandon plastic spill-free slide jar (Thermo Scientific, Waltham, MA) containing 0.01 M citrate buffer, pH 6.2, with 0.002 M EDTA. After cooling for 20 min, slides were washed in PBS and permeabilized in 1.0% Triton X-100 in PBS for 30 min. Slides were rinsed again in PBS and sections of interest were encircled with a PAP pen. Sections were blocked in 10% normal donkey serum, 1.0% BSA, and 0.03% Triton X-100 diluted in PBS for 30 min. Incubation with the primary antibody mix was performed at 4°C overnight in a wet chamber followed by incubation with the mix of fluorophore conjugated secondary antibodies at room temperature for 2 hr in a wet chamber protected from light. Both primary and secondary antibodies were diluted in 1% NDS, 1% BSA, and 0.03% Triton X-100. After washing, slides were mounted with anti-fading agent Gel/Mount (Biomeda, Foster City, CA, PA).

Primary antibodies used were the following: anti-insulin (1∶300, Abcam), anti-glucagon (1∶400, Abcam), anti-somatostatin (1∶400, Abcam) and anti-PBEF/Nampt (1∶200, custom-made rabbit a-N412(s431) lot 852829/N412-2 (7-21-10) or custom-made rabbit aPBEF/Nampt C18/T57(2-04-2011) [22]. Corresponding secondary antibodies were conjugated with DyLight 488 (1∶400, Jackson ImmunoResearch Laboratories Inc.), Alexa 555 (1∶400, Molecular Probes) and Alexa 647 (1∶400, Molecular Probes). Non-specific staining was identified using samples exposed to secondary antibodies only.

Images were obtained on an Olympus Fluoview confocal microscope or a Nikon C1Si or a C1Plus confocal microscope. Images were acquired using 10X –100X objectives (depending on the experiment), and analyzed using FluoView or Ps Adobe Photoshop CZ4 software. The border of each islet was identified with beta-cell staining against insulin. All images were corrected by subtracting background fluorescence. Analysis of endocrine/exocrine Nampt staining required increase in brightness of each picture by 20% in order to obtain reliable values from the fetal tissues, which had very low levels of staining. This manipulation was applied to all images analyzed for the endocrine/exocrine ratio levels. Co-localization studies were completed by double-staining sections with antibodies against Nampt and insulin or Nampt and glucagon. In order for a cell to be identified as positive for either protein, the appropriate fluorescence had to be at least 2 times above the background level.

### Glucose Stimulated Insulin Secretion (GSIS)

Approximately 350 IEQ human islets with a reported purity and viability of approximately 85%–95% were placed in 24 well PET 1um Millicell insert plates (Millipore, Carrigtwohill, Ireland). Islets were cultured overnight in CMRL1044 10% fetal calf serum, prior to the experiment. There were 3 wells per treatment group and 7 different islet preparations used. The GSIS was conducted by exposing islets to Krebs Ringer Buffer (119 mM NaCL, 4.7 mM KCL, 25 mM NaHCO3, 2.5 mM CaCL2, 1.2 mM MgSO4, 1.2 mM KH2PO4, 0.2% BSA) with low glucose (2.2 mM) for 1 hour followed by exposure to Krebs Ringer Buffer with high glucose (20 mM) for 1 hour. In some experiments inhibitors of insulin secretion, diazoxide (0.5 mM) and nifedipine (20 nM) from Sigma (Saint Louis, MO) were added to the Krebs Ringer Buffer 20 mM glucose solution. Supernatants were collected to determine insulin and eNampt content by ELISA and islets were collected and snapped frozen for RNA and protein assays. Nampt and insulin protein was determined by ELISA based assays from AdipoGen International (San Diego, CA) and ALPCO Immunoassays (Salem, NH) respectively. Assays were performed according to the manufacturer's instructions.

### Islet Extracts for Western Blot

Islets were washed in phosphate-buffered saline (PBS) and homogenized by RIPA protein extraction buffer (50 Mm TrisHCL pH 7.4, 150 mM NaCl, 2 mM EDTA, 1%NP-40, 0.1% SDS). Protein concentrations were measured using Micro BCA Protein Assay Kit (Pierce, Rockford, IL). Islet extracts were prepared for electrophoresis by heating at 95°C for 3 min in SDS gel-loading buffer (5× stock: 0.0625 M Tris, pH 6.8).

### Islet Culture Supernatant Preparation for Western Blot

Supernatant was collected from GSIS and stored in 500ul aliquots at −80°C until ready for analysis. A buffer exchange/concentration was performed (25 mM Tris, 75 mM NaCl, pH 7.5 solution) using Amicon Ultra 30K devices (30,000 NMWL (Nominal Molecular Weight Limit)) (Millipore, Billerica, MA) to a volume of 50ul. Albumin was removed from the samples using the Swell Gel Blue Albumin Kit (Pierce Thermo Scientific, Rockford, IL) according to the manufacturer's instructions. The volume of the sample was brought up to 300ul with low salt buffer (25 mM Tris, 75 mM NaCl pH 7.5) and immunoprecipitated using an anti-Nampt antibody (mouse antibody, anti-Nampt (Visfatin/PBEF) OMNI379, AdipoGen, Incheon, Korea). To prepare the immunopreciptation reaction, 2.5ug of anti-NAMPT antibody, 300ul of sample, and 100ul of Protein A Trisacryl Bead Slurry (Pierce Biotechnology, Rockford, IL) were added to a 1.5 ml tube. The reaction was incubated overnight at 4°C with agitation (for efficient mixing). Each reaction was spun down at 2500×g for 3 min. The supernatant from the immunoprecipitation reaction was removed and stored in a 1.5 ml tube. The bead pellet was washed three times with 25 mM Tris, 75 mM NaCl buffer, pH 7.5. NAMPT was eluted off the beads with 50ul 4 M MgCl_2_ for 5 minutes. The elution step was performed twice and elutes were pooled from each sample. Finally, the 4 M MgCl_2_ was exchanged for 75 mM NaCl using the Amicon Ultra 30K devices (EMD Millipore, Billerica, MA). To prepare samples for Western Blot analysis, the samples were heated to 95°C for 3 min in SDS gel-loading buffer (5× stock: 0.0625 M Tris, pH 6.8, 25% glycerol, 5.3% mercaptoethanol, 2% SDS, and 0.01% bromophenol blue). Western blot results were normalized by using equal amounts of supernatant (500 ul).

### Western Blot

Proteins were separated on a 10% PAGE non-commercially prepared gel with 25 mM Tris, 192 mM glycine, and 0.1% SDS running buffer. Equal amounts of total protein (40 µg) were loaded in each lane. Molecular weight markers Kaleidoscope Prestained Standards (Bio-Rad, Hercules, CA) and Magic Mark XP Western Blot Standard (Invitrogen, Carlsbad, CA) were used for visualization and to verify the size of antigen, respectively. After electrophoresis, the proteins from the gel were transferred to a Immun-blot PVDF membrane 0.45 µm (Bio-Rad, Hercules, CA) using 25 mM Tris, 192 mM glycine, 0.1% SDS, and 20% methanol transfer buffer. Blots were blocked with 5% nonfat dry milk diluted in 0.1 M PBS 0.05% Tween 20 (PBST) for 1 hour and probed with primary antibodies (1∶3000 dilution AdipoGen, Incheon, Korea) against NAMPT overnight at 4°C. After washing in 0.1 M PBS 0.05% Tween 20, the membrane (islet extract) was probed with rabbit anti-actin (1∶500 dilution Santa Cruz Biotechnology, Inc. (sc-81178) Santa Cruz, California) for 1 hour as a protein loading control. After washing in 0.1 M PBS 0.05% Tween 20, blots were incubated for 1 hour with secondary antibody, horseradish peroxidase-conjugated goat anti-rabbit IgG (1∶25,000 dilution Jackson ImmunoResearch, West Grove, PA). After washing in 0.1 M PBS 0.1% Tween, bound antibodies were detected using Amersham ECL Plus western blotting reagents (GE Healthcare Life Sciences, Pittsburgh, PA).

### eNampt enzymatic activity assay

Supernatants collected from 4 different islet cultures in Krebs Ringer Buffer 20 mM glucose underwent albumin depletion, buffer exchange, and concentrated from 2 ml to 50ul as described above. The reaction was carried out in triplicate as previously described [23]. The reaction volume (100ul) contained 50 mM Tris-HCL, 10 mM MgCl2, 50 mM Nicotinamide, 0.2 mM PRPP, 2.0 mM ATP, Nmnat 10ug/ml (R&D Systems, Minneapolis, MN), 1.5% ethanol, and 10ug/ml alcohol dehydrogenase. In some cases recombinate human Nampt (AdipoGen International, San Diego, CA) was used in place of superntant eNampt for positive controls, and for negative controls the substrate, nicotinamide, was omitted. Unless stated otherwise, all reagents were from Sigma (St. Louis, MO). The reaction was carried out for 45 minutes at 37°C followed by the addition of NAD extraction buffer. NAD+/NADH content was determine by using NAD+/NADH quantification kit (Biovision Research Products, Mountain View, CA) following manufacturers instructions.

### In Situ Nampt Binding Assays

Nampt binding to islet cell surface proteins was evaluated using a modified histological assay described from McCudden et al [24]. Isolated human islets were paraformaldehyde fixed paraffin embedded, or snapped frozen, or air dried on slides. In some cases cryosections or air dried cells were post-fixed with ice cold acetone. Paraffin sections were dewaxed, rehydrated and in some cases antigen retrieval was employed (citrate buffer/heat or 1N HCL). All sections or cells were washed in Hanks Balanced Salt Solution +0.5% Bovine serum albumin prior to incubate at room temperature for 90 minutes with human recombinant Nampt histidine tagged or unlabeled Nampt (3–12 ug/ml), (Axxora LLC, San Diego, CA). Post fixation was performed at room temperature after incubation with the primary antibody (60% acetone/3% formalin for 45 seconds). Tissues were then incubated with a second antibody, 6-histidine epitope tag horseradish peroxidase conjugated antibody (Novus Biologicals, Littleton, CO; 1∶200-500 dilutions) at room temperature for 2 hours. Lastly, the chromogen substrate (3-Amino-9-Ethylcarbazole (AEC)) was applied with positive staining noted as a red color.

### Statistics

Unless otherwise noted, data are presented as means ± SEM. Statistical analyses used Student's t test or ANOVA as appropriate. Significance was defined as p<0.05.

## Results

### Nampt gene expression in non-diabetic human pancreas and isolated islets

**Figure 1:**
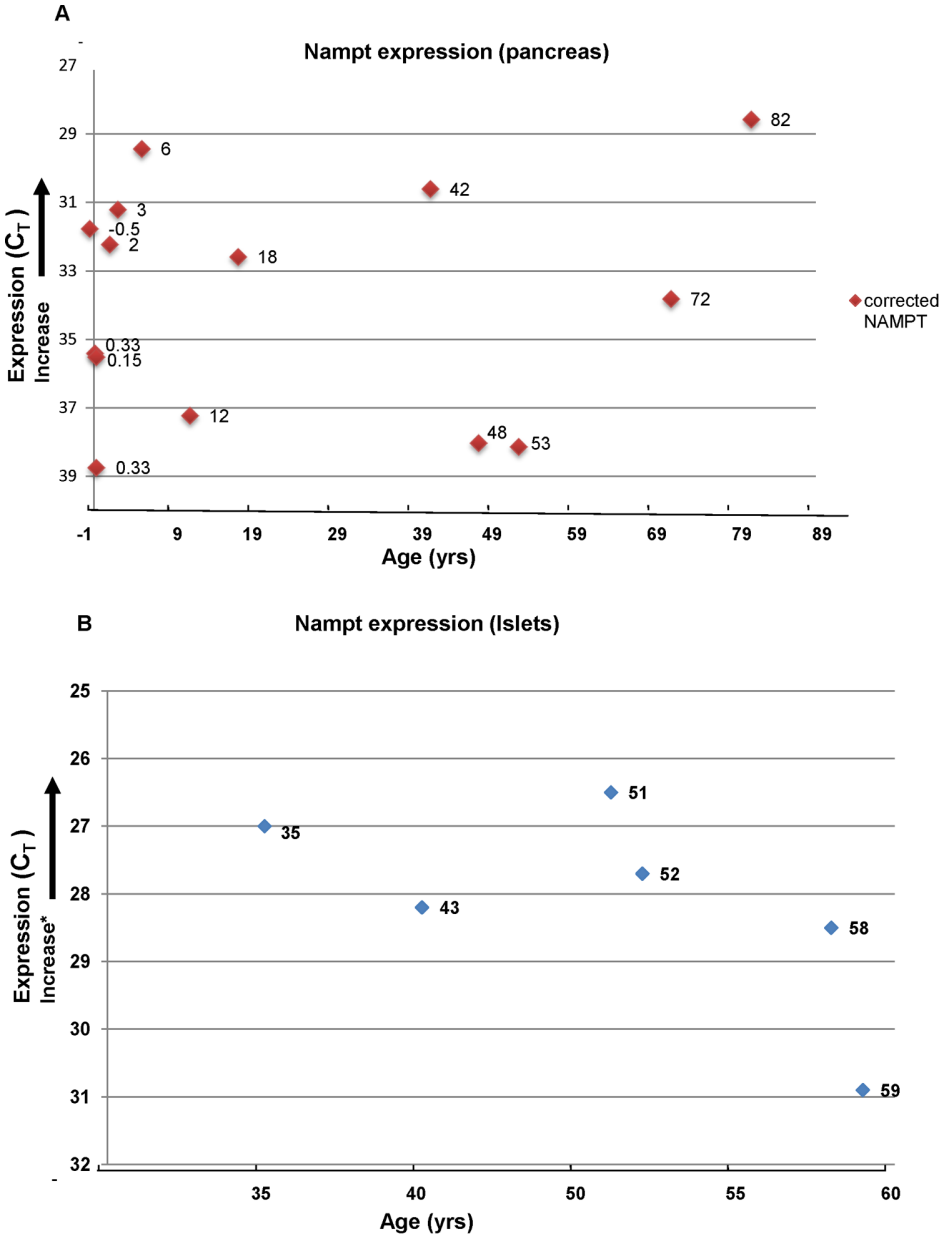
Quantification of Nampt mRNA . Total RNA was isolated from **A.** human pancreatic tissue or **B.** isolated human islets. NAMPT mRNA was quantified using a Nampt specific Taqman assay (Applied Biosystems/Life Technologies, Carlsbad, CA.) according to the manufacturer's instructions and normalized with GUSB. *Note: The Y axis uses C_T_ values (C_T_ is the threshold cycle of detection), thus increased target mRNA results in earlier detection by qRT-PCR (i.e., a smaller C_T_).

Nampt gene expression has not been characterized in human pancreatic tissue or islets. We used qRT-PCR with TaqMan probes to examine endogenous expression of Nampt in human pancreatic tissue and isolated islets. Nampt message was detected in both human pancreatic tissue samples (Figure 1A) and in isolated human islets (Figure 1B). We evaluated a wide age span (fetal to 82 years) and saw substantial variability in expression of Nampt. Interestingly, there did not appear to be a relationship between Nampt expression and age. Likewise in our isolated islets, although the sample size was small, no correlation with age was evident.

### Nampt protein expression in non-diabetic human pancreas and isolated islets

**Figure 2:**
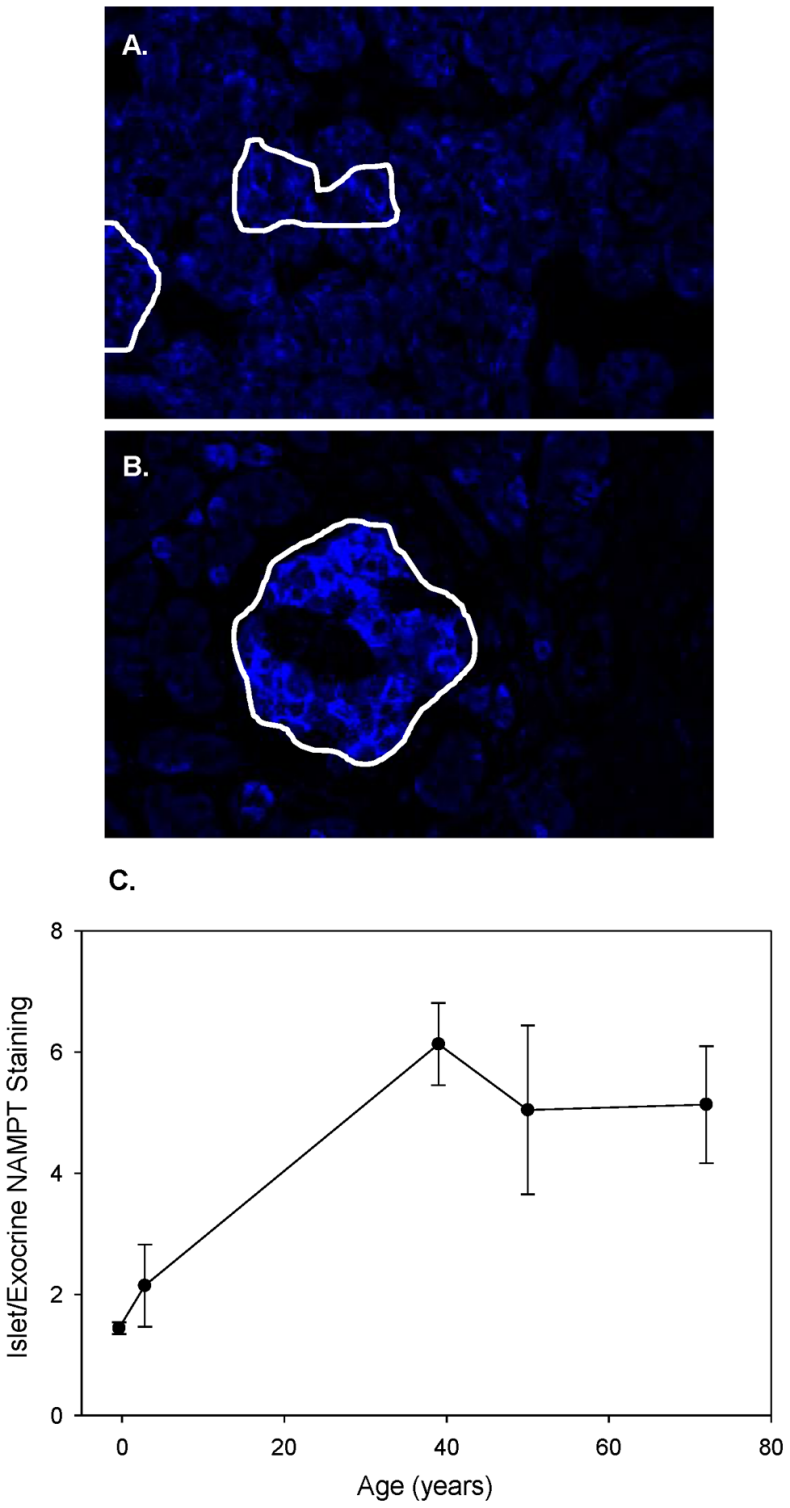
Protein pattern of Nampt in human islets changes with age. The difference in Nampt staining in endocrine and exocrine cells is clear. A: Fetal pancreatic tissues showed nearly equal Nampt staining levels in endocrine (within white circled regions) and exocrine tissue. B: In contrast, tissue from a 39 year old shows bright Nampt staining within the islet. C: The ratio of endocrine to exocrine pixel intensity illustrates the change with age. Of note, total image brightness was increased by 20% for every pancreatic image analyzed for figure C in order to visualize the low levels of Nampt staining in the fetal tissues.

Protein levels of Nampt were identified in both the exocrine and endocrine cells using immunofluorescene staining techniques. The exocrine staining was more prominent in the samples from young children, starting with fetal tissues (Fig. 2A). In fact there was little difference in the amount of Nampt staining in the exocrine versus endocrine cells in the fetal pancreata examined. However, in adulthood, the location of Nampt was predominantly in endocrine cells and that localization was stable throughout life (Figs. 2B). The ratio of endocrine/exocrine fluorescence intensity increased early in life, but stabilized in adulthood (Fig. 2C).

**Figure 3:**
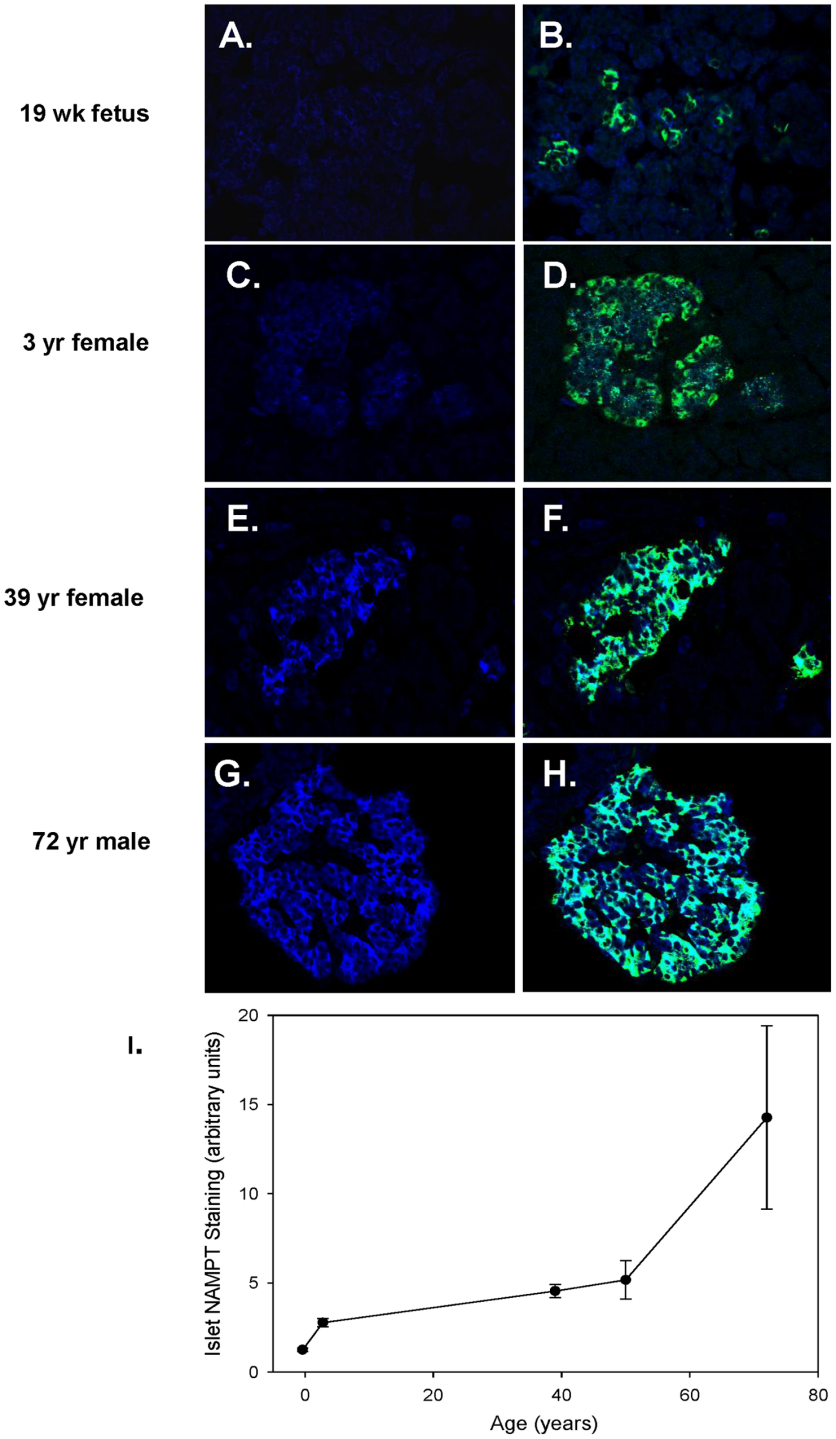
Protein levels of Nampt in human islets are greater with age. Examples of Nampt (blue) and insulin (green) immunofluorescence co-staining in islets from donors varying from 19 weeks gestation to 72 years old. **A–D:** In fetus and young children Nampt staining was weak with little co-localization with insulin in beta cells. **E–H:** In adults, Nampt staining was stronger and more localized to beta cells. **I:** Analysis of the Nampt pixel intensity illustrates the change with age.

Within the islets, the majority of Nampt staining was identified in the beta cells, co-localizing with insulin rather than glucagon. Figure 3 provides images of Nampt staining at different donor ages (Figs. 3A, C, E, & G) and co-localization with insulin (Figs. 3B, D, F, & H). From these example images, it is clear that the intensity of NAMPT immuno-staining increased with age Fig. 3I).

**Figure 4:**
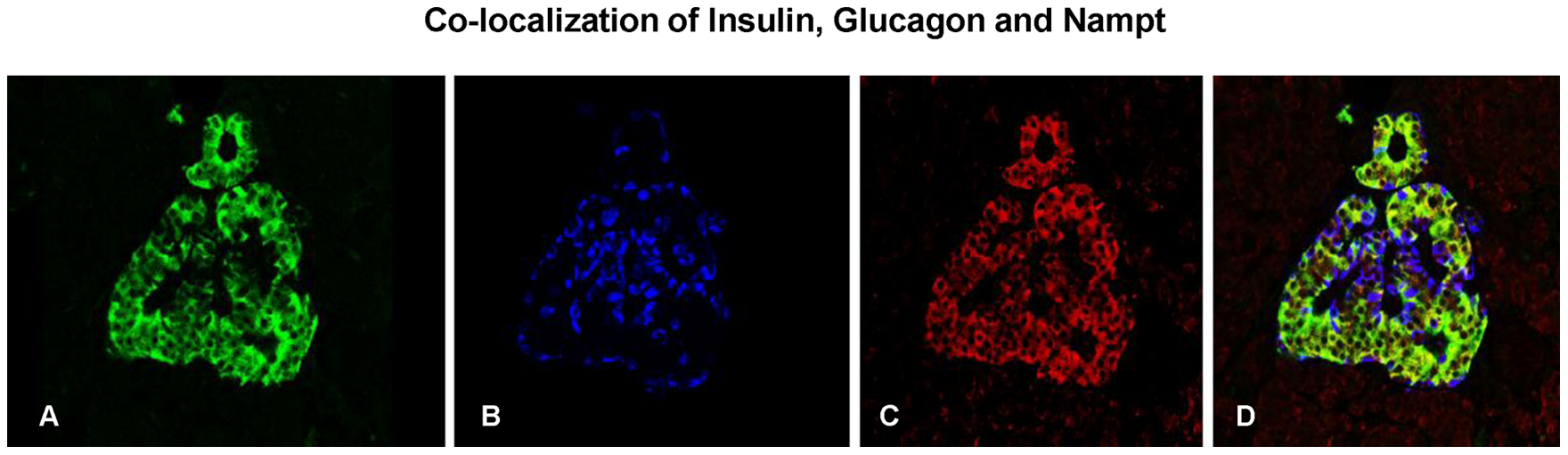
Co-localization of insulin, glucagon, and Nampt. Immunofluorescence image of an islet from an adult male stained for insulin, glucagon and Nampt. **A:** insulin staining (beta cells) using anti-insulin antibody (green). **B:** Glucagon was identified in the same islet (alpha cells) using anti-glucagon antibody (blue). **C:** Nampt was identified in the same islet using anti-Nampt antibody (red) and is found in both the islet and surrounding exocrine tissue. **D:** Overlap of all 3 images shows that the majority of Nampt co-localizes with insulin in beta cells.

Some co-localization with glucagon occurred within the alpha cells. However, the majority of Nampt staining was identified in beta cells. Figure 4 illustrates the predominant co-localization of Nampt with insulin staining (green) and less with glucagon staining (blue). On average there was 2.52±0.22 times more Nampt staining in the beta cells compared to alpha cells.

### Glucose Stimulated Insulin and Nampt Secretion

**Figure 5:**
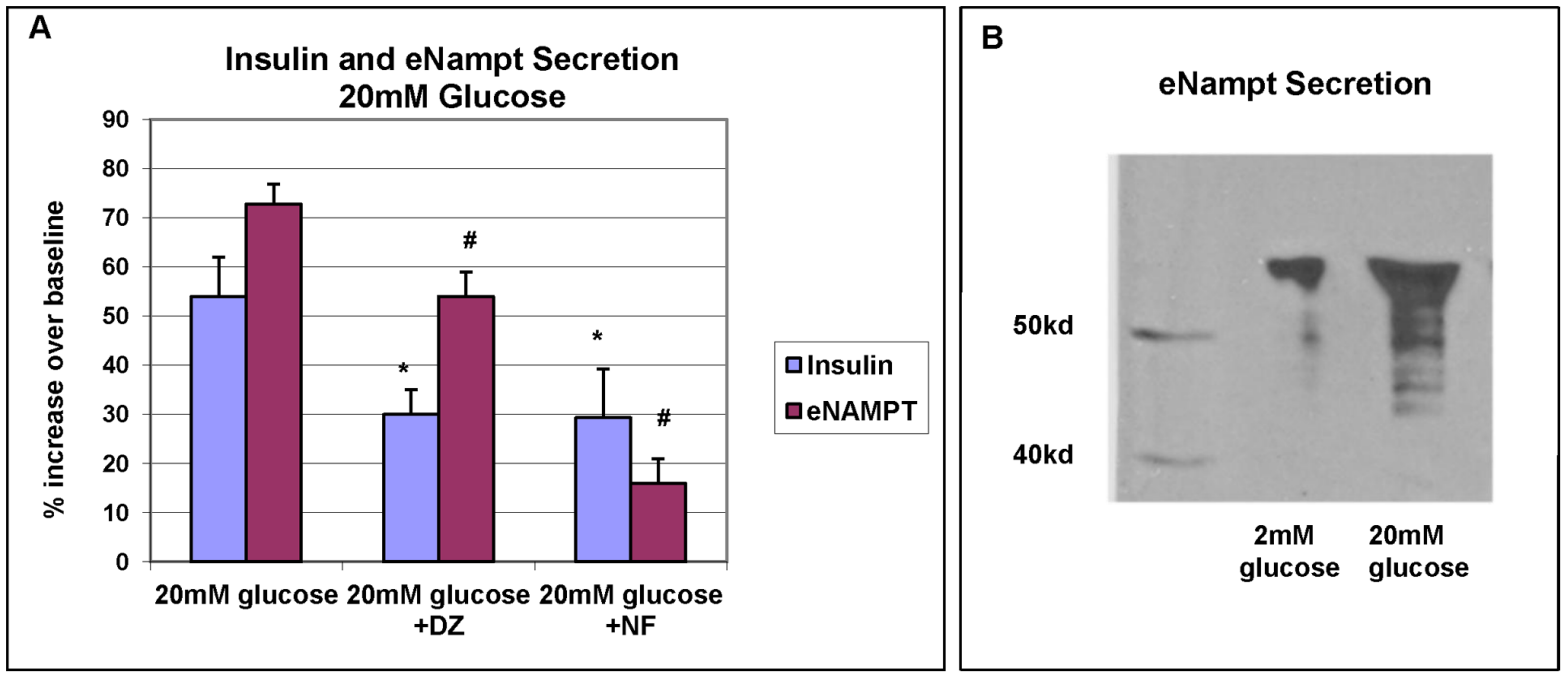
Glucose stimulated insulin and eNampt secretion in human islets. A static glucose-stimulated insulin secretion test was performed. **A:** 20 mM glucose stimulated insulin and eNampt secretion as measured by ELISA. Percent change from baseline (2.2 mM glucose) is shown. Depolarization inhibitors, Diazoxide (DZ) and Nifedipine (NF) attenuated secretion. Results are the mean values from 7 separate experiments.^* #^ p<0.05; student T test. **B:** Higher levels of eNampt were detected in 20 mM glucose culture supernatant compared to 2.2 mM glucose by Western blot analysis. Shown is a representative blot from 6 separate experiments.

A static incubation GSIS test was performed on 7 different groups of isolated human islets. As expected, exposure to 20 mM glucose resulted in a robust release of insulin (50–60% increase) over baseline. The 7 islets groups had a median basal insulin secretion level of 3679 uIU/ml (interquartile range 3105–4660uIU/ml) and a median 20 mM glucose stimulated insulin secretion level of 7227uIU/ml (interquartile range 5612–10100uIU/ml). The robust release of insulin was attenuated (∼25%) by inhibitors of beta cell depolarization, diazoxide and calcium channel antagonist, nifedipine (Fig. 5A). Supernatants from the static incubation were also assayed for the presence of eNampt by ELISA. Surprisingly, not only was eNampt detected in the supernatant, but protein levels were significantly higher in supernatants tested from the 20 mM glucose cultures compared to 2.2 mM glucose cultures (an average increase of 70–75%) (Fig. 5A). The 7 groups of islets had a median basal eNampt secretion level of 302 pg/ml (interquartile range 75–414 pg/ml) and a median 20 mM glucose stimulated eNampt secretion level of 560 pg/ml (interquartile range 97–1579 pg/ml). This suggests that secretion of eNampt by islets is glucose sensitive. In addition, secretion of eNampt was attenuated by modifying beta cell depolarization using diazoxide (∼20% decrease) or nifedipine (50–60% decrease). The glucose stimulated increase in eNampt secretion was confirmed by western blot analysis (Fig. 5B). A viability assay was performed to verify that the increase in eNampt levels found in the supernatant was due to secretion and not leakage from dying cells. The viability of the cells within the islets was measured during the GSIS test using live/dead fluorophores, YO-PRO-1 and propidium iodide. Samples of islets were collected from 2.2 mM cultures and 20 mM cultures with cell viability measured at 81.3±3.6% and 81.8±3.2% respectively (data not shown).

### The effects of glucose on Nampt expression in human islets

**Figure 6:**
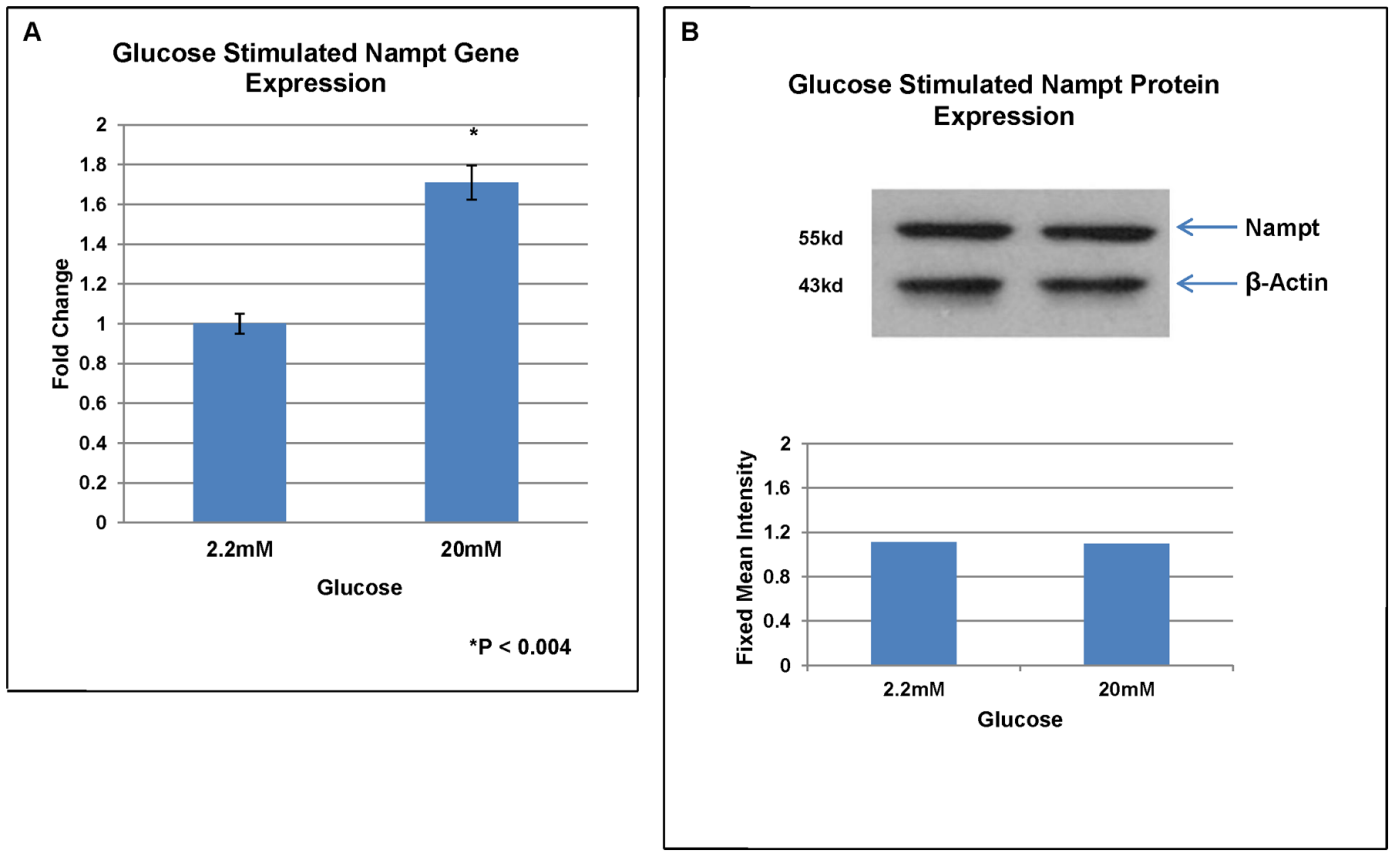
The effects of glucose on Nampt gene and protein expression levels in human islets. Total RNA or protein was isolated from islets treated for 1 hour in 20 mM glucose. **A:** Nampt gene expression was upregulated in the presence of 20 mM glucose compared to control (2.2 mM glucose) by qRT-PCR. Each bar represents the mean fold change normalized to β-actin from five separate experiments.*P<0.05; student T test **B:** Nampt protein content did not increase in the presence of 20 mM compared to 2.2 mM glucose by western blot analysis. Shown is a representative blot from 6 separate experiments.

To determine if glucose modulates Nampt gene and protein expression, islets were collected after 1 hour exposure to 2.2 mM or 20 mM glucose and processed for RNA and protein isolation. There was a significant increase in Nampt mRNA levels (1.71 fold) in islets exposed to 20 mM glucose compared to 2.2 mM by RT-PCR analysis (Fig. 6A). In contrast, no significant differences in Nampt protein expression was detected between islets incubated in 2.2 mM and 20 mM glucose by western blot analysis (Fig. 6B).

### Enzymatic activity of islet eNampt

**Figure 7:**
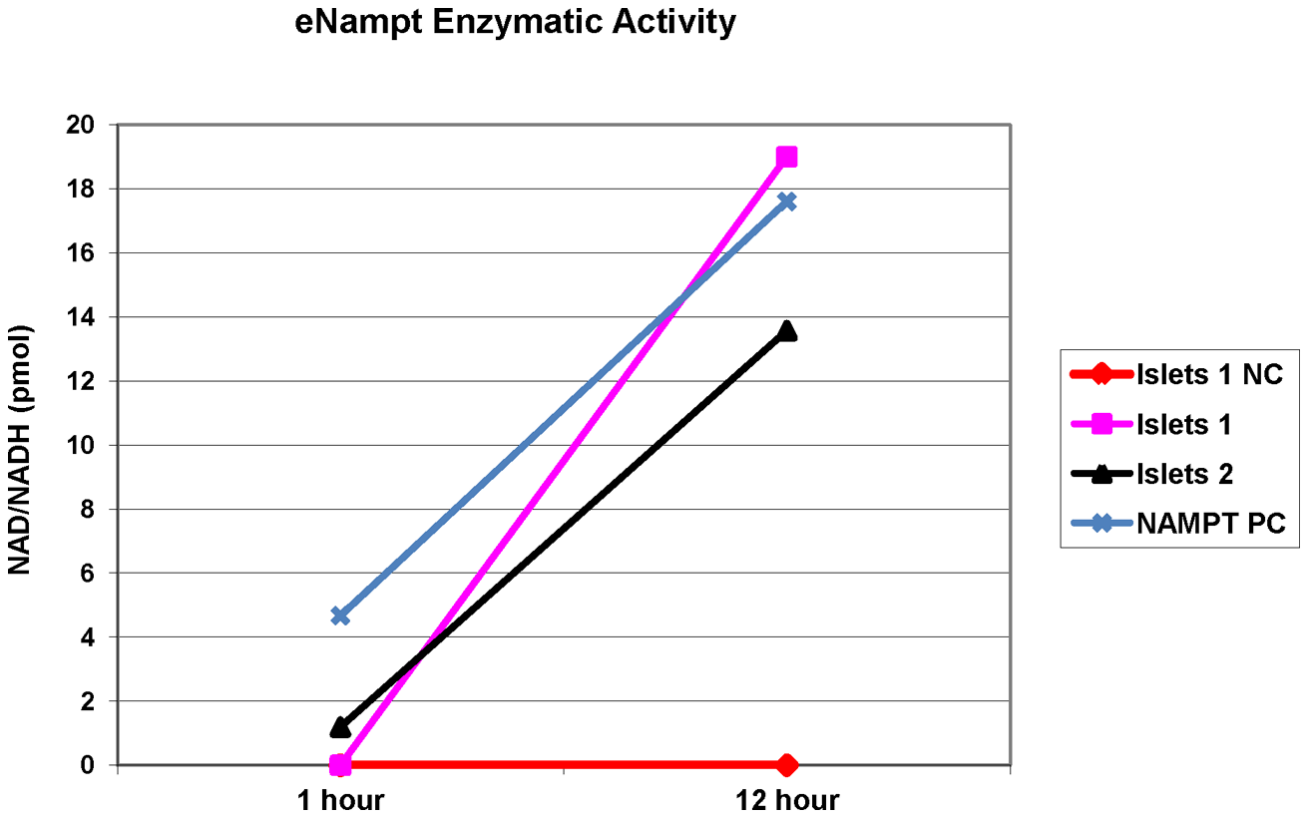
Enzymatic activity of eNampt secreted by human islets. eNampt was concentrated from supernatant collected from islet cultures after 1 hour exposure to 20 mM glucose. An enzyme-coupled fluorometric assay was carried out in triplicate using supernatant from separate islet donors, a positive control (a human recombinant Nampt (Nampt PC)) and a negative control (NC). Supernatant collected from 4 islets groups and the positive control, showed enzymatic activity with a rise in NAD+/NADH content over time while the negative control did not show activity. Two out of 4 experiments with identical results are shown.

To determine if eNampt secreted by human islets is enzymatically active, we performed a modified enzyme-coupled fluorometric assay using islet eNampt from 20 mM glucose supernatants from GSIS experiments. The assay results showed enzymatic activity in the islet supernatant and the positive control (recombinant human Nampt) with increasing production of NAD+/NADH over time (Fig. 7). Omitting the substrate, nicotinamide, resulted in no biosynthesis of NAD+/NADH in the samples tested (Fig. 7, NC).

### eNampt binding on human islets

To determine if eNampt binds to cell surface receptors on islets we performed an in situ binding assay using histidine-tagged human Nampt. After 1 hour exposure to 20 mM glucose, islets were collected and either air dried, frozen, or formalin fixed paraffin embedded. Dried whole mount islets, frozen or paraffin sectioned islets were tested for Nampt/receptor binding under a variety of conditions including different fixatives, antigen retrieval methods, and post-fixation methods (to stabilize ligand/receptor binding). Under all conditions and antibodies tested we did not find specific positive staining to indicate that Nampt binds to the surface of human islet cells (Table 2).

**Table 2:** Nampt in situ binding assay – summary of conditions tested.

Islet sections	Fixation	Antigen retrieval	Nampt Hist-tag or unlabeled	Post-fixation	His-tag antibody	Substrate (AEC) and results
frozen	none	no	90min RT	yes	30min -1hr RT	negative
frozen	acetone	no	90min RT	yes	30min -1hr RT	negative
frozen	PFA	no	90minRT	yes	30min -1hr RT	negative
paraffin	PFA	HCL	90 min RT	yes	30min -1hr RT	negative
paraffin	PFA	Citrate/heat	90 min RT	yes	30min -1hr RT	negative
Whole mount (air dried)	PFA	no	90 min RT	yes	30min -1hr RT	Non-specific staining
Whole mount (air dried)	acetone	no	90 min RT	yes	30min -1hr RT	Non-specific staining

PFA, paraformaldehye; HCL, hydrochloric acid; RT, room temperature.

## Discussion

Nampt has ubiquitous tissue distribution with the highest expression found in peripheral blood lymphocytes, heart, liver, lungs, and lowest in the brain and pancreas [25], [26]. Expression of Nampt in human islets has not been described as previous studies have primarily focused on the effects of eNampt on islet function. Our goal was to characterize islet specific Nampt expression, secretion, and regulation by glucose.

In this study we present evidence that Nampt is expressed in exocrine and endocrine tissue during fetal development. After birth, Nampt expression decreases in exocrine tissue but remains in endocrine cells, predominately beta cells. Nampt protein expression appears to increase in beta cells with age. There was no correlation with age and Nampt gene expression but that may be due to the variable amount of endocrine cells present in tissue samples tested. We also show that human islets secrete eNampt and secretion is regulated by glucose. Similar findings have been reported with human primary adipocytes where eNampt secretion increased by 50% in human primary adipocytes after exposure to 11.1 mM glucose in culture [27]. In contrast, others report no effect of glucose on Nampt release in hepatocytes or leucocytes ex vivo [26], [28]. The different results most likely reflect tissue specificity regarding the regulation of Nampt expression and release.

In our studies eNampt secretion was partially dependent on membrane depolarization as inhibitors of membrane depolarization such as diazoxide and nifedipine (L-type voltage-gated calcium channel blocker) reduced eNampt secretion. This suggests that mechanisms required for eNampt release from islets are similar to insulin, requiring membrane depolarization and Ca++ influx for exocytosis. The release of eNampt by HIB-1B brown adipocyte cell line, 3T3-L1 adipocytes and CHO cell line is reportedly through a non-classical pathway [1], [29]. Investigators reported that an inhibitor of protein secretion, brefeldin A, acting through the golgi-ER complex, did not have an effect on eNampt secretion. Similar findings were found in hepatocyte cultures where constitutive release of eNampt was not affected by brefeldin A, monensin, or glibenclamide [28]. Since NAMPT lacks a signal sequence for secretion, it has been suggested that the presence of eNampt is due to cell lysis [30], [31]. In our experiments the presence of eNampt in the supernatant was not due to leakage from dying cells as we performed a viability assay and did not see differences in islet cell viability over the course of the experiment.

In addition to eNampt secretion, we found 20 mM glucose increased Nampt gene expression but protein levels remain constant in human islets at the time point analyzed. We speculate that the protein levels may appear constant because the newly synthesized Nampt is replacing the mature Nampt that is co-secreted with insulin in response to glucose. Translation efficiency and/or varying degrees of degradation rates of mRNA and protein may also play a role in mRNA-protein discordance.

In addition to glucose, insulin may act as an autocrine factor regulating eNampt release. It has been reported that insulin can suppress eNampt secretion in mammary epithelial cell and primary adipocyte cell cultures [27], [32]. While we did not investigate the effects of insulin on eNampt secretion, we did observe that eNampt and insulin secretion trended towards a negative inverse relationship suggesting that insulin may have a role in regulating eNampt secretion (data not shown). While a strong negative inverse relationship was noted (Pearson correlation r = −0.75), the data did not reach statistical significance. We acknowledge that we had a small sample size and the experiment needs to be repeated using a larger number of donors. Variability in the function of isolated human islets has been previously reported [33], [34]. We noted that high responder islets, with abundant insulin release in response to 20 mM glucose, had low levels of eNampt secretion. In contrast islets that were low responders, with modest insulin release in response to 20 mM glucose, had significantly higher eNampt secretion. It is possible that when intracellular NAD+ levels drop due to metabolic stress and insulin secretion is impaired, beta cells tried to compensate by secreting eNampt in order to generate NMN. NMN would be taken up by beta cells to generate NAD+, increase Sirtuin 1 activity and restore insulin secretion. Perhaps the addition of nicotinamide and/or NMN during a GSIS test might restore insulin secretion in the low responder islet group. The mechanisms that drive the unique interplay between eNampt and insulin in regulating beta cell response to glucose have yet to be elucidated.

The role(s) of eNampt in beta cell function is still evolving. Here we report that human islet eNampt has enzymatic activity. As previously discussed, there is compelling evidence that extracellular enzymatic activity of this protein is important in supporting insulin secretion by generating NMN. However this is only apparent when beta cells are under metabolic stress or deficient in Nampt [1], [19]. Therefore, eNampt may have a compensatory role in supporting insulin secretion when beta cell dysfunction is present. Our results suggest that the enzymatic activity of eNampt is not required for beta cells to respond to glucose under normal conditions as NMN was not generated during the GSIS (no substrate in medium), yet in many cases we detected a robust insulin release in response to 20 mM glucose. In addition to supporting insulin release NMN was reported to reduce the expression of pro-inflammatory cytokines, pro-apoptotic, and oxidative stress related genes in isolated mouse islets exposed to inflammatory conditions [19]. Taken together the evidence suggests that the major role of eNampt is to generate NMN, to help correct beta cell dysfunction and protect beta cells from metabolic and oxidative stress.

Besides enzymatic activity, eNampt may induce intracellular signaling by binding to cell surface receptors. While somewhat controversial, there are reports that Nampt activates insulin receptors leading to receptor phosphorylation. It was reported that Nampt induced tyrosine phosphorylation of insulin receptors and insulin receptor substrates in primary human osteoblast cells, leading to increased glucose uptake, proliferation, and type 1 collagen production [4]. Brown et al. reported insulin receptor phosphorylation and subsequent ERK 1/2 activation in beta-TC6 cells after exposure to Nampt [3]. Interestingly, Brown et al also reported similar findings using NMN in place of Nampt. It should be noted that most commercially available cell culture media contains nicotinamide, the substrate for Nampt. An argument could be made that Nampt-stimulated insulin receptor activity may be due, in part, to the generation of NMN in these experiments. The most definitive data showing Nampt binding to insulin receptors was by Fukuhara et al. [5]. These investigators performed competitive binding assays showing that Nampt and insulin had similar binding equilibrium dissociation constants (K_D_) to insulin receptors. However this paper was retracted due to the discovery that not all recombinant Nampt protein preparations bound and activated insulin receptors [35]. In contrast others found that Nampt did not induce insulin receptor phosphorylation or downstream AKT activity when tested on several preadipocyte cell lines [1]. Using his-tagged Nampt protein, we completed experiments, exhaustively searching for binding of his-tagged Nampt on isolated human islets using immunohistochemistry techniques. We did not find any specific staining under the various conditions tested. It may be that our conditions were not optimal or the methods employed were not sensitive enough to detect Nampt/receptor binding. Additional studies are needed to conclusively determine if eNampt binds to a membrane bound receptor on beta cells.

In summary, we show that Nampt is expressed in both exocrine and endocrine tissue early in life. However, in adulthood, Nampt expression is found mostly in beta cells and this pattern of expression is stable throughout life. In addition human beta cells secrete enzymatically active eNampt that is regulated by glucose. eNampt secretion, like insulin secretion, is dependent on membrane depolarization.
